# Salicylic Acid Reduces Salinity Stress in Barbados Cherry Irrigated with Oilfield Water in Semiarid Brazil

**DOI:** 10.1021/acsomega.5c07698

**Published:** 2025-10-02

**Authors:** Reginaldo Gomes Nobre, Kaila Maria Pereira de Carvalho, Guilherme da Silva Sales, Maria do Socorro Medeiros de Souza, Antônio Gustavo de Luna Souto, Luiz Fernando de Sousa Antunes

**Affiliations:** † Department of Science and Technology, 74384 Federal Rural University of the Semi-Arid (UFERSA), 59780000 Caraúbas, Rio Grande do Norte, Brazil; ‡ Federal Rural University of the Semi-Arid (UFERSA), Center of Agrarian Sciences, 59625900 Mossoró, Rio Grande do Norte, Brazil

## Abstract

The Barbados cherry (*Malpighia emarginata* DC) is a fruit crop of significant economic, social, and nutritional importance, particularly in Northeast Brazil. Its sustainable cultivation in semiarid regions depends on irrigation due to the negative water balance during most months. Given water scarcity, strategies such as using lower-quality water, including brackish water, wastewater, and oilfield produced water (OPW), have been investigated. These water sources, combined with proper irrigation management and salicylic acid (SA), can mitigate abiotic stresses like salinity. This study evaluated Barbados cherry rootstock production under irrigation with synthetic OPW and SA concentrations at the Federal Rural University of the Semi-Arid Region, Caraúbas—RN, Brazil. A randomized block design with a 5 × 4 × 2 factorial arrangement and four replications was used. Treatments included five OPW dilutions in supply water (SW): D1 (100% SW), D2 (75% SW + 25% OPW), D3 (50% SW + 50% OPW), D4 (25% SW + 75% OPW), and D5 (100% OPW), combined with four SA concentrations (0, 0.8, 1.6, and 2.4 mM) and two genotypes (*Junco* and *Crioula*). Results show that OPW, combined with SA, enables irrigation in semiarid regions, reducing improper OPW disposal and promoting water conservation. The average SA concentration of 1.3 mM alleviated saline stress effects up to 2.63 dS m^–1^, enhancing biomass production and seedling quality. The OPW-D3 dilution (50% SW + 50% OPW) was most effective for seedling morphophysiology, with *Junco* showing the best quality and *Crioula* exhibiting greater saline stress tolerance. These findings highlight the potential of combining OPW and SA as a sustainable strategy for Barbados cherry seedling production under saline conditions.

## Graphical abstract

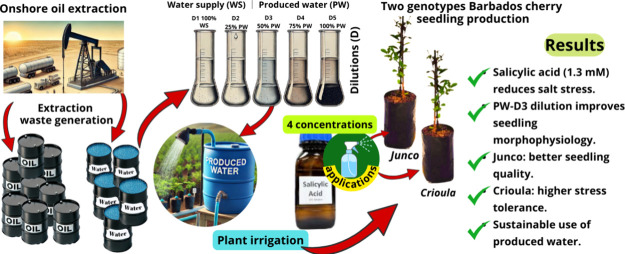

## 1 Introduction

Agriculture in the Brazilian Semi-Arid region plays a fundamental role in food security and socioeconomic development. However, adverse climatic factors, such as low rainfall and high temperatures, pose challenges to the sustainability of agricultural production, making irrigation an indispensable resource for the sector’s viability.^1^ The growing demand for food, driven by population growth, exacerbates pressure on water resources, especially given the scarcity of quality freshwater for expanding cultivated areas. In this context, the use of alternative water sources, such as brackish and wastewater, has become an increasingly common practice in the region.^2^

To ensure the sustainability of agricultural production, it is essential to adopt strategies that reduce environmental impacts and improve the efficiency of natural resource use. Irrigation not only meets the water demand of crops but also mitigates climate variability, enabling year-round production.^3^ However, water availability has become an increasingly critical limiting factor, especially in arid and semiarid regions where agriculture is highly dependent on irrigation.^4^ In this scenario, the reuse of lower-quality water, such as treated effluents, agricultural drainage water, and brackish water, emerges as a viable and strategic alternative for maintaining agricultural activity,^5–7^ aligning with the United Nations Sustainable Development Goals (SDGs),^8^ particularly SDG 2 – Zero Hunger and Sustainable Agriculture.

Among alternative water sources, oilfield produced water (OPW) has garnered increasing interest due to its significant availability in oil-producing regions, often located near agricultural areas.^9^ OPW is a byproduct of oil and natural gas extraction, characterized by large volumes and a complex chemical composition, including dispersed oils, dissolved organic compounds, dissolved solids, metals, and radioisotopes.^10,11^ In this sense, irrigation with water produced from petroleum in the long term, if not treated to rigorous and specific levels to remove heavy metals and radioisotopes, may present a risk of contamination of the soil and underground sources of drinking water, soil fauna and agricultural crops.^12,13^

In Brazil, OPW production reached approximately 3.99 million barrels per day in 2017, representing about 1.5 barrels of water for each barrel of oil extracted.^14^ Proper management of this water is a challenge for the oil industry, both due to the high costs of disposal and treatment and the potential environmental impacts.^15,16^ Currently, the main destinations for OPW include reinjection into wells, controlled disposal, and reuse in various activities, including agricultural irrigation.^11^ The feasibility of agricultural use of OPW depends on effective management strategies to mitigate its potential negative effects. Generally, this water has high salt content, which can affect soil structure and impair plant growth by inducing physiological stress and reducing crop yield.^5^ To minimize these impacts, several practices have been studied, including the selection of salt-tolerant species, dilution with other water sources, and the use of substances that alleviate saline stress, such as salicylic acid, hydrogen peroxide, and proline.

Salicylic acid (SA), in particular, has been widely studied for its ability to induce tolerance to saline stress and other abiotic stresses in plants, regulating essential biochemical and physiological processes such as ion uptake and balance, activation of antioxidant enzymes, and modulation of endogenous hormones and gene expression.^17–19^ SA is a powerful phytohormone and signaling molecule that significantly influences various physiological and biochemical processes during plant growth and development. Beyond its well-documented role in mitigating abiotic stress, SA is also crucial in enhancing plants’ immune responses to biotic stresses. This is achieved through intricate signaling pathways, molecular interactions, and synergistic relationships with other phytohormones, such as jasmonic acid (JA), ethylene (ET), and abscisic acid (ABA).^19^

This strategy may be especially relevant for fruit crops adapted to the conditions of the Brazilian Semi-Arid region, such as the Barbados cherry, also known as acerola (*Malpighia emarginata* DC., widely cultivated in the Northeast, the country’s main production hub.^20^ According to the Census of Agriculture,^21^ Brazil produced 60,966 tons of Barbados cherry, with the Northeast region contributing 47,607 tons, or approximately 78.1% of the national total. Pernambuco State stands out as the largest producer within the region, accounting for 21,351 tons, or nearly 45% of the Northeast’s production. In addition to its high productive potential and adaptability to local edaphoclimatic conditions, the Barbados cherry is highly valued for its exceptional vitamin C content and other bioactive compounds, serving as an important source of income for both family farming and agribusiness.^22^

In this context, this study aimed to evaluate the production of Barbados cherry seedlings irrigated with different dilutions of OPW, combined with exogenous application of salicylic acid at varying concentrations. The research seeks to contribute to advancing knowledge on the sustainable use of OPW in agriculture, exploring strategies that can mitigate its adverse effects and expand its potential as an alternative water resource for semiarid regions.

## 2 Materials and Methods

### 2.1 Experimental Site and Conditions

The research was conducted from July 28, 2023, to January 15, 2024, under protected environment conditions (shade house) in an experimental area at the Multidisciplinary Center of Caraúbas, Federal Rural University of the Semi-Arid Region (UFERSA), located in the western region of Rio Grande do Norte, Brazil. The geographical coordinates are 05°46′23″ S and 37°34′12″ W, with an altitude of 144 m. The region’s climate is classified as BSh according to the Köppen classification, indicating a hot semiarid climate.^23^

### 2.2 Experimental Design and Treatments

The treatments were arranged in a 5 × 4 × 2 factorial scheme with four replications, each plot consisting of one plant. The treatments included five dilutions of synthetic oilfield produced water (OPW) in local supply water (SW): D1 (100% SW), D2 (75% SW + 25% OPW), D3 (50% SW + 50% OPW), D4 (25% SW + 75% OPW), and D5 (100% OPW), combined with four concentrations of salicylic acid (SA) (0, 0.8, 1.6, and 2.4 mM) and two Barbados cherry genotypes (*Junco* and *Crioula*). The different OPW dilutions had the following salinity levels: D1 (0.47 dS m^–1^), D2 (1.26 dS m^–1^), D3 (2.06 dS m^–1^), D4 (2.63 dS m^–1^), and D5 (3.26 dS m^–1^), with pH values of D1 (7.11), D2 (7.43), D3 (7.68), D4 (7.75), and D5 (7.79).

### 2.3 Plant Material

Two Barbados cherry genotypes were used: the *Junco* cultivar, known for its high fruit production and widely cultivated in the Northeast, with fruits containing over 3000 mg of ascorbic acid per 100 g of pulp,^24^ and the *Crioula* genotype, whose seeds were obtained from the municipality of Caraúbas-RN. The *Crioula* genotype is adapted to local edaphoclimatic conditions and shows good fruit production. Due to the lack of information in the literature regarding salicylic acid concentrations for Barbados cherry, the values were chosen based on the research of Silva et al.,^25^ who conducted a study on soursop under saline stress.

### 2.4 Preparation of Synthetic Oilfield Produced Water (OPW)

The synthetic OPW was prepared using local supply water (UFERSA/Caraúbas-RN), adding specific salts according to the concentrations established by Figueiredo (2014) for 1000 L of solution: aluminum chloride (1.162 g), borax (3.88 g), boric acid (2.519 g), calcium chloride (425.74 g), calcium sulfate (55.89 g), iron chloride (0.695 g), manganese sulfate (0.522 g), magnesium chloride (557.05 g), potassium chloride (89.46 g), sodium chloride (609.5 g), and zinc sulfate (0.222 g). These authors analyzed OPW from different oil wells in the Potiguar Basin and determined the average chemical composition, which showed characteristic values of 3.26 dS m^–1^ electrical conductivity, along with predominant elements including calcium (169.87 mg L^–1^), chloride (687.47 mg L^–1^), magnesium (65.76 mg L^–1^), and sodium (242.07 mg L^–1^). After preparation, the OPW dilutions were stored in 90 L plastic containers, properly protected to prevent evaporation and contamination.

The synthetic OPW was formulated based on the average chemical composition of produced water from the Potiguar Basin, as determined by Figueiredo^26^ from an analysis of 85 samples collected from 23 wells across five distinct production zones. This approach was adopted for two primary reasons: first, it provides a representative benchmark of the water quality in the region of interest, ensuring the relevance of our findings to local conditions. Second, access to real OPW samples from oilfield companies is highly restricted and typically contingent upon complex project agreements, making a synthetic analogue the most feasible and reproducible option for controlled experimentation. While we recognize that real OPW exhibits significant spatial and temporal variability in its ionic composition, salinity, and organic content, the use of a defined synthetic standard allows for the isolation of salinity effects and ensures the reproducibility of our results.

### 2.5 Sowing and Transplanting Barbados Cherry Genotypes

Sowing was carried out in polyethylene trays, with two seeds per cell, planted at a depth of 1 cm. After 50 days, seedlings were transplanted into 1150 mL plastic bags, with two seedlings per bag. The bags were filled with soil collected from a depth of 0–30 cm in the municipality of Caraúbas-Rio Grande of Norte State, mixed with 2% (by weight) of cured cattle manure. The bags were placed on wooden racks at a height of 0.2 m above the ground to facilitate management. Excess water was drained through holes at the bottom of the bags. After 50 days of transplanting, thinning was performed, leaving only the most vigorous plants per bag.^27^

### 2.6 Chemical and Physical-Hydric Analysis of Growing Media

Following the methodology proposed by Teixeira et al.,^28^ the chemical and physical-hydric characteristics of the growing media used in the experiment were analyzed before the start of the study (Table 1).

**Table 1:** Chemical and Physical-Hydric Characteristics of the Growing Media Used for Sowing Barbados Cherry, UFERSA, 2024^a^

parameter	unit	value
sand	g kg^–1^	446
silt	g kg^–1^	411
clay	g kg^–1^	143
textural classification		loamy
EC	dS m^–1^	0.68
pH (H_2_O)		6.03
organic matter	g kg^–1^	37
P	mg dm^–3^	134.2
K^+^	cmolc dm^–3^	0.87
Na^+^	cmolc dm^–3^	0.15
Ca^2+^	cmolc dm^–3^	17.11
Mg^2+^	cmolc dm^–3^	1.14
Al^3+^	cmolc dm^–3^	0
(H^+^+ Al^3+^)	cmolc dm^–3^	0.58
sum of bases	cmolc dm^–3^	19.27
cation exchange capacity	cmolc dm^–3^	19.27
V (base saturation)	%	97
m (aluminum saturation)	%	0
exchangeable sodium percentage	%	1

a Organic matter: wet digestion Walkley-Black method; Ca^2+^ and Mg^2+^ extracted with 1 mol L^–1^ KCl at pH 7.0; Na^+^ and K^+^ extracted using 1 mol L^–1^ NH_4_OAc at pH 7.0; Al^3+^ and (H^+^ + Al^3+^) extracted using 1 mol L^–1^ CaOAc at pH 7.0; EC – electrical conductivity of the saturation extract at 25 °C; pH – pH of the saturation extract of the growing media.

### 2.7 Irrigation and Phytosanitary Control

During the experimental period, the+ growing media was maintained at moisture levels close to field capacity. Until 73 days after transplanting (DAT), the plants were irrigated with local supply water, which had an electrical conductivity of 0.47 dS m^–1^. After this period, the plants were irrigated daily with oilfield produced water (OPW), based on the principle of drainage lysimetry. The volume applied in each irrigation was determined by the difference between the applied volume and the drained volume the following day, with this difference equivalent to the volume of water required for the soil to reach its maximum water retention capacity (field capacity). In this process, 20 randomly selected bags were equipped with plastic bags to collect the drained water.

Phytosanitary control was carried out preventively and/or curatively in response to the incidence of pests and diseases, as well as manual removal of weeds. A commercial product (CP) with the active ingredient Imidacloprid (10% w/v) was used, with the prepared solution consisting of 1 mL of CP per liter of water.

### 2.8 Application of Salicylic Acid (SA)

The salicylic acid (SA) concentrations were applied exogenously at 70, 79, 87, 95, 103, and 111 DAT by spraying on the adaxial and abaxial leaf surfaces to ensure complete leaf wetting. A volume of 3 mL per plant was initially applied, increasing to 5 mL as the plants grew. Applications were made at 4:30 PM using a spray bottle and a support to prevent drift to other treatments.

### 2.9 Data Collection and Analysis

The effects of the different treatments were evaluated based on growth variables, biomass, physiological parameters, and seedling quality of Barbados cherry. Growth variables, including number of leaves (NL), plant height (PH), and stem diameter (SD), were assessed at 122 DAT. For NL, only leaves with fully expanded blades were counted. Both SD and PH were measured above the growing media level: SD at 3 cm above the neck, and PH from the neck to the insertion point of the newest leaf.

At 98 DAT, physiological variables were measured using a portable infrared carbon dioxide analyzer (IRGA), model LCorp-SD from BioScientific (Hoddesdon, UK). The analyzed variables included intercellular CO_2_ concentration (*Ci*; μmol m^–2^ s^–1^), stomatal conductance (*gs*; mol H_2_O m^–2^ s^–1^), and CO_2_ assimilation rate (*A*; μmol CO_2_ m^–2^ s^–1^). Measurements were taken on the fourth leaf from the apex to the base of the branch.

At the end of the experimental period (122 DAT), the final morphological evaluation was conducted. Plants were cut at the growing media level, and the roots and shoots (leaves and stems) were separated. These were placed in labeled paper bags and dried in an oven with air circulation at 65 °C until constant weight was achieved. The leave dry mass (LDM), stem dry mass (SDM), root dry mass (RDM), and total dry mass (TDM) per plant was determined using a precision scale.

Seedling quality was determined at the end of the experiment (122 DAT) using the Dickson Quality Index (DQI), calculated according to Dickson et al.:^29^

$$\mathrm{DQI}=[\mathrm{TDM}÷(\frac{\mathrm{PH}}{\mathrm{SD}})]+(\frac{\mathrm{SDM}}{\mathrm{RDM}})$$

where:

- DQI = Dickson Quality Index,
- TDM = Total dry mass (g),
- PH = Plant height (cm),
- SD = Stem diameter (mm),
- SDM = Shoot dry mass (g),
- RDM = Root dry mass (g).

### 2.10 Statistical Analysis

The means of the variables were subjected to analysis of variance (ANOVA) using the F-test at 0.05 and 0.01 probability levels. Data related to SA concentrations were analyzed using regression studies, and the means of qualitative factors (OPW dilutions and Barbados cherry genotypes) were compared using Tukey’s test (1 and 5% probability). The statistical software SISVAR version 5.6 ^30^ was used for analysis.

## 3 Results and Discussion

### 3.1 Stomatal Conductance (gs), Intercellular CO_2_ Concentration (Ci), and CO_2_ Assimilation Rate (A)

By evaluating the summary of the analysis of variance (Table 2), a significant effect of the interaction between the factors oilfield produced water (OPW) × salicylic acid (SA) concentrations and the isolated factor different Barbados cherry genotypes on stomatal conductance (*gs*) is identified. The intercellular CO_2_ concentration (*Ci*) was significantly affected by the interaction between OPW × Barbados cherry genotypes (GA). Additionally, there was a significant isolated effect of the different OPW dilutions on the CO_2_ assimilation rate (A).

**Table 2:** Summary of the Analysis of Variance for Stomatal Conductance (*gs*), Intercellular CO_2_ Concentration (*Ci*), and CO_2_ Assimilation Rate (*A*) of Barbados Cherry Genotypes Seedlings under Irrigation with Oilfield Produced Water (OPW) Dilutions and Different Salicylic Acid (SA) Concentrations

	mean squares		
source of variation	*gs*	*Ci*	*A*
oilfield produced water (OPW)	27194.672^c^	23501.995^c^	86.079^c^
salicylic acid concentrations (SA)	2349.913^b^	3170.242^a^	13.825^a^
linear regression	4668.727^c^	4786.039^a^	0.556^a^
quadratic regression	626.828^a^	1974.165^a^	12.00^a^
Barbados cherry genotypes (BCg)	2706.272^b^	455.692^a^	27.656^a^
interaction (OPW × SA)	2381.713^c^	2064.254^a^	11.858^a^
interaction (OPW × BCg)	922.979^a^	6981.049^b^	10.407^a^
interaction (SA × BCg)	776.699^a^	3110.962^a^	3.99^a^
interaction (OPW × SA × BCg)	1390.982^a^	3451.403^a^	8.547^a^
blocks	512.330^a^	5182.536^a^	2.98^a^
CV (%)	24.82	32.46	25.44

a Not significant.

b Significant at *p* ≤ 0.01.

c Significant at *p* ≤ 0.05; CV: coefficient of variation.

The different Barbados cherry genotypes differed significantly in terms of stomatal conductance, with the highest value (103.98 mol H_2_O m^–2^ s^–1^) observed in the *Junco* genotype (Figure 1A). This value was 7.91% higher than that of the *Crioula* genotype, indicating that the *Junco* genotype has greater efficiency in regulating stomatal opening, promoting more effective gas exchange and enhancing adaptation to environmental conditions. This may benefit plant growth and photosynthetic efficiency.^31^

**Figure 1:**
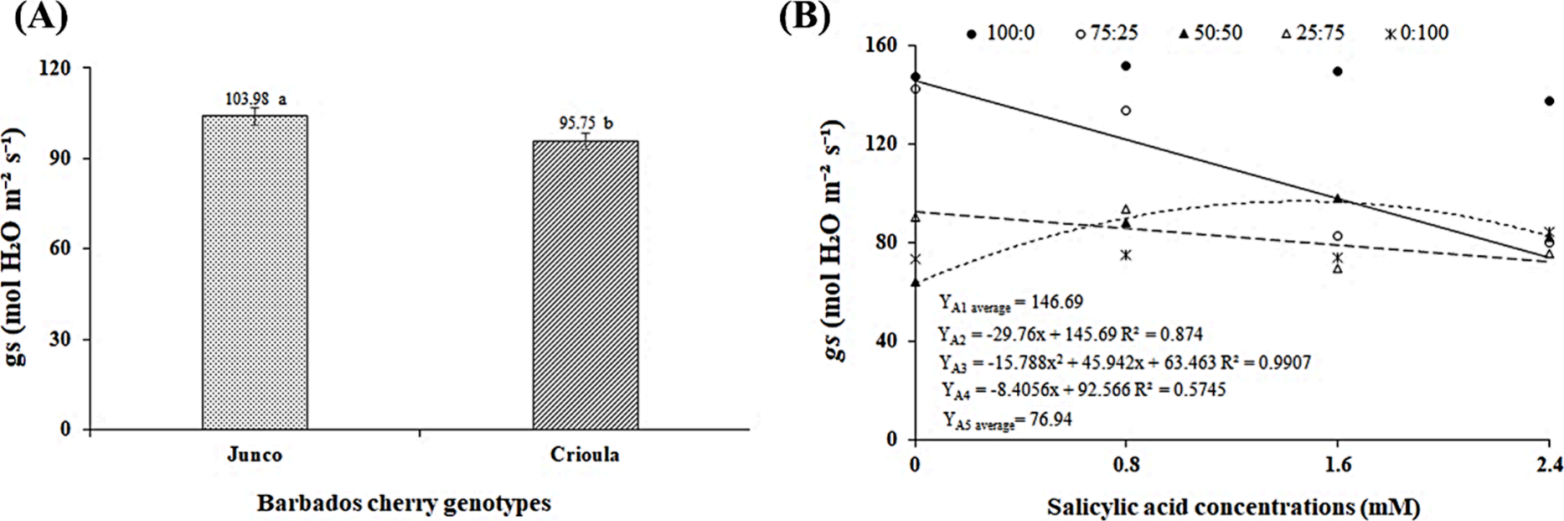
Stomatal conductance (*gs*) as a function of different Barbados cherry genotypes (A) and the interaction between the factors oilfield produced water (OPW) and salicylic acid (SA) concentrations (B). Dilutions: D1 (100% supply water (SW)), D2 (75% SW + 25% OPW), D3 (50% SW + 50% OPW), D4 (25% SW + 75% OPW), and D5 (100% OPW). Means with the same letters do not differ from each other according to Tukey’s test (*p* ≤ 0.01).

Stomatal conductance (*gs*) was also affected by the interaction between the factors OPW × SA. According to the regression eqs (Figure 1B), a linear behavior was observed in plants irrigated with OPW-D2 and OPW-D4, with decreases of 20.43 and 9.08% per unit increase in SA concentration, respectively. This resulted in reductions of 49.02 and 21.79% in seedlings that received the highest SA concentration compared to those that did not receive SA. In plants under the OPW-D3 dilution, a quadratic response was observed, with the highest value (96.85 mol H_2_O m^–2^ s^–1^) obtained at an SA concentration of 1.5 mM. This indicates that the *gs* of seedlings irrigated with the 50% SW + 50% OPW dilution was positively influenced by the application of salicylic acid, which may be related to stress signaling triggered by SA application. This increases the production of secondary metabolites, which reduce the osmotic potential of the roots to levels lower than those caused by salt accumulation in the soil, improving water and nutrient uptake and potentially increasing stomatal opening.^32^

For intercellular CO_2_ concentration (*Ci*), a significant interaction was observed between the OPW dilutions × Barbados cherry genotypes (Table 2). According to the mean comparison test (Figure 2A), the highest Ci in the *Junco* genotype occurred in plants irrigated with OPW-D1, which did not differ statistically from OPW-D2 and was 38.51% higher than in plants under OPW-D5. In the *Crioula* genotype, the highest *Ci* (202.83 μmol CO_2_ m^–2^ s^–1^) was observed in seedlings under OPW-D1, which did not differ statistically from plants under OPW-D3, OPW-D4, and OPW-D5, surpassing plants under OPW-D2 by 33.07%. Furthermore, the genotypes differed only in the OPW-D2 dilution, with *Junco* being 23.3% higher than *Crioula* in this dilution. However, overall, the *Crioula* genotype was more tolerant to the saline effects of OPW, as there was almost no significant difference across the different treatments. In the case of the *Junco* genotype, the difference was attributed to carbon consumption by RuBisCO in the Calvin cycle, which reduces the carbon present in the substomatal chambers. Damage to this process reduced carbon consumption, resulting in an energy imbalance caused by salt accumulation in the plant, intensifying the production of reactive oxygen species.^33^

**Figure 2:**
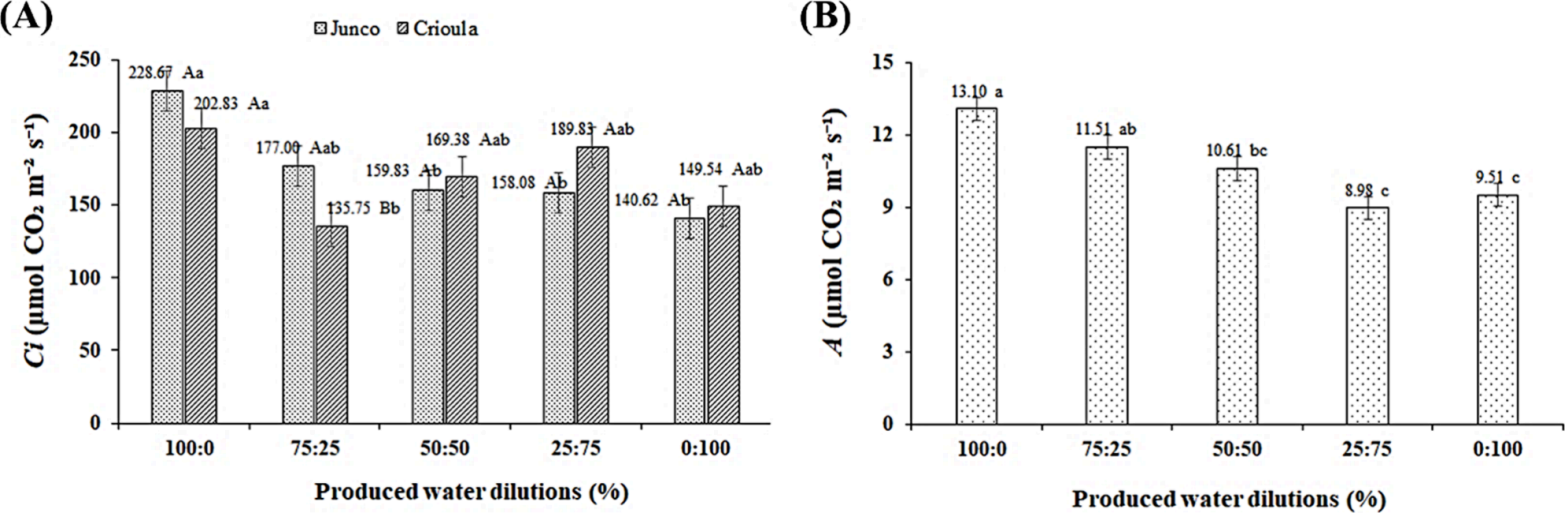
Intercellular CO_2_ concentration (*Ci*) as a function of the interaction between the factors oilfield produced water (OPW) and Barbados cherry genotypes (A), and CO_2_ assimilation rate (*A*) as a function of OPW dilutions (B). Dilutions from left to right: D1 (100% supply water (SW)), D2 (75% SW + 25% OPW), D3 (50% SW + 50% OPW), D4 (25% SW + 75% OPW), and D5 (100% OPW). Means with the same letters do not differ from each other according to Tukey’s test (*p* ≤ 0.05).

The increase in salinity in the irrigation water, present in the different OPW dilutions, significantly affected the CO_2_ assimilation rate (A). According to the mean comparison test (Figure 2B), the highest value was observed in plants irrigated with 100% supply water (OPW-D1), which did not differ from OPW-D2 and was 31.45% higher than seedlings irrigated with OPW-D4, which showed the lowest value for this variable. This may have occurred due to the reduction in stomatal conductance (*gs*) and the consequent decrease in CO_2_ diffusion, which tend to negatively impact net photosynthesis.^34^

### 3.2 Growth Variables: Number of Leaves (NL), Plant Height (PH), and Stem Diameter (SD)

The summary of the analysis of variance (Table 3) reveals a significant effect of the interaction between the studied factors (oilfield produced water (OPW) × Barbados cherry genotypes (BCg) on number of leaves (NL) and plant height (PH), as well as the interaction (OPW × salicylic acid (SA) concentrations) on PH and stem diameter (SD).

**Table 3:** Summary of the Analysis of Variance for Number of Leaves (NL), Plant Height (PH), and Stem Diameter (SD) of Barbados Cherry Genotypes under Irrigation with Oilfield Produced Water (OPW) Dilutions and Different Salicylic Acid (SA) Concentrations at 122 Days after Transplanting (DAT)

	mean squares		
source of variation	NL	PH	SD
oilfield produced water (OPW)	146.8309^c^	110.5566^c^	0.3058^b^
salicylic acid concentrations (SA)	23.2018^a^	11.5052^a^	0.2423^a^
linear regression	5.5594^a^	7.775^a^	0.4329^a^
quadratic regression	10.6760^a^	0.164^a^	0.2933^a^
Barbados cherry genotypes (BCg)	401.1639^c^	439.5358^c^	0.4420^a^
interaction (OPW × SA)	34.6605^a^	49.5923^a^	0.2985^a^
interaction (OPW × BCg)	73.2937^b^	119.3579^c^	0.1861^a^
interaction (SA × BCg)	3.3100^a^	20.4304^a^	0.1108^a^
interaction (OPW × SA × BCg)	25.9167^a^	33.4993^a^	0.3856^a^
blocks	240.9762^c^	945.8313^c^	1.5886^c^
CV (%)	12.09	9.77	6.39

a Not significant.

b Significant at *p* ≤ 0.01.

c Significant at *p* ≤ 0.05; CV: coefficient of variation.

The number of leaves (NL) was significantly affected by the interaction between the factors OPW × BCg. According to the mean comparison test (Figure 3A), the highest leaf production in the *Junco* cultivar occurred in plants under the OPW-D1 dilution, which did not differ statistically from OPW-D3 and OPW-D4 and was 17.58% higher than in plants under OPW-D5. For the *Crioula* genotype, the highest NL was observed in plants under OPW-D2, which did not differ statistically from OPW-D1 and OPW-D3 and surpassed OPW-D5 by 12.19%. Furthermore, the *Junco* genotype outperformed *Crioula* in terms of NL when subjected to the OPW-D1, OPW-D3, and OPW-D4 dilutions, while the *Crioula* genotype achieved the highest NL under the OPW-D2 dilution. Based on the results, it can be inferred that the genotypes tolerated the salts in the irrigation water up to the OPW-D4 dilution (*Junco*) and OPW-D3 dilution (*Crioula*), corresponding to an average salinity of 2.35 dS m^–1^. This indicates that in the OPW-D5 dilution (100% OPW), which had an electrical conductivity of 3.52 dS m^–1^, osmotic stress reduced water and nutrient uptake, affecting leaf production in the plants.^35^

**Figure 3:**
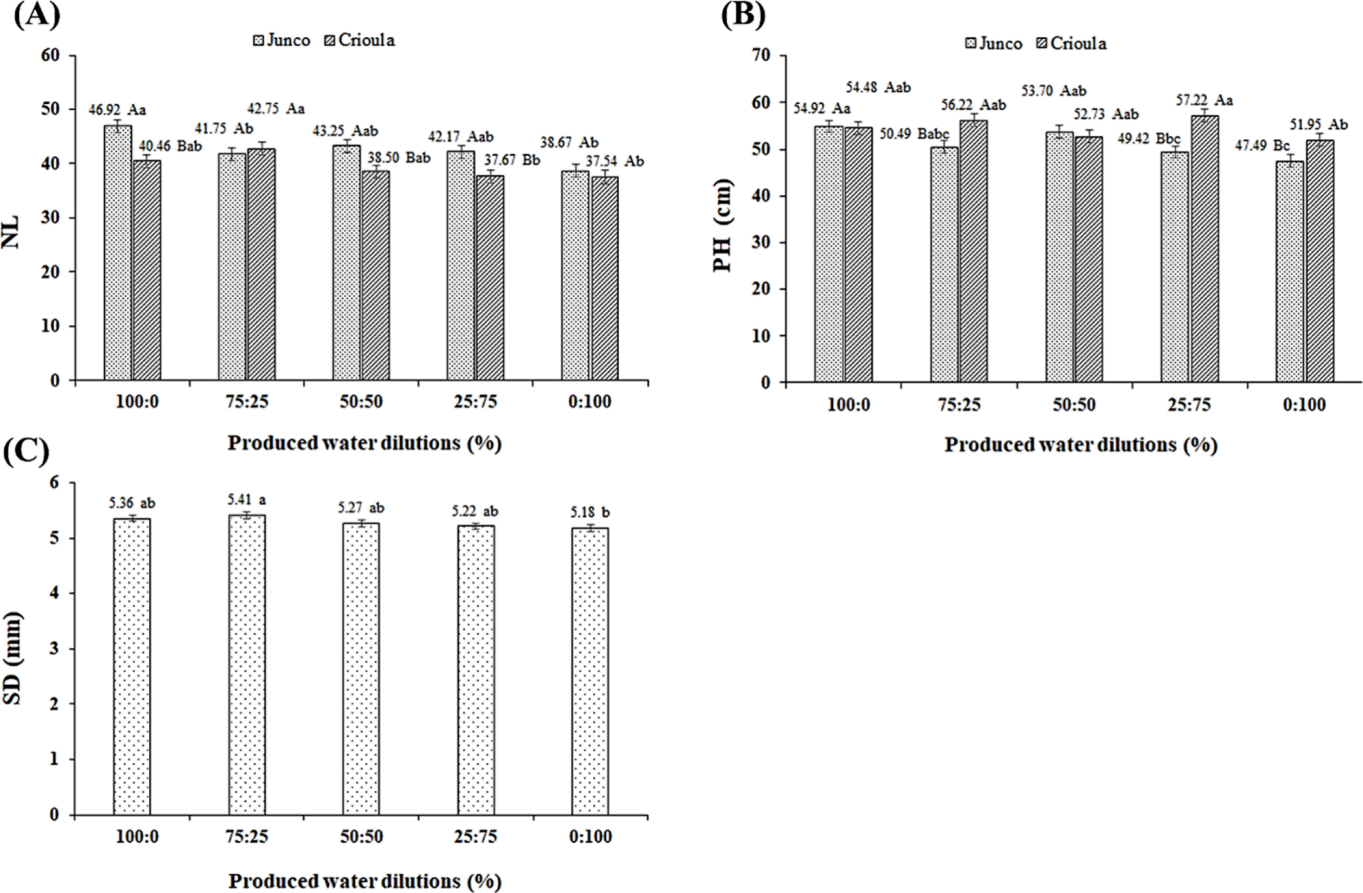
Number of leaves (NL) (A) and plant height (PH) (B) as a function of the interaction between the factors oilfield produced water (OPW) and Barbados cherry genotypes, and stem diameter (SD) (C) as a function of OPW dilutions, all at 122 days after transplanting (DAT). Dilutions from left to right: D1 (100% supply water (SW)), D2 (75% SW + 25% OPW), D3 (50% SW + 50% OPW), D4 (25% SW + 75% OPW), and D5 (100% OPW). Means with the same letters do not differ from each other according to Tukey’s test (*p* ≤ 0.01 for NL and PH, and *p* ≤ 0.05 for SD).

The interaction between the factors OPW × BCg also significantly affected plant height (PH). According to the mean comparison test (Figure 3B), the highest PH in the *Junco* cultivar was observed under OPW-D1 (100% supply water), which did not differ statistically from OPW-D2 and OPW-D3 and was 13.53% higher than in plants under OPW-D5 (100% OPW). For the *Crioula* genotype, the greatest height was observed in seedlings under OPW-D4 (25% SW + 75% OPW), which did not differ statistically from OPW-D1, OPW-D2, and OPW-D3 and was 9.21% higher than in plants under OPW-D5. This indicates that seedlings of both genotypes had good tolerance to the salts in the irrigation water in terms of height (Figure 3B), as even when irrigated with the OPW-D5 dilution (3.52 dS m^–1^), they showed an average reduction of 11.37%. Additionally, the *Crioula* genotype outperformed *Junco* under irrigation with OPW-D2, OPW-D4, and OPW-D5, with no statistical difference under OPW-D1 and OPW-D3, likely due to the genetic characteristics of each plant material.

In Figure 3C, it is observed that the increase in salinity in the irrigation water significantly affected stem diameter (SD). The lowest value (5.18 mm) was observed in plants irrigated with OPW-D5, which was 3.36% lower than in seedlings under OPW-D1 (5.36 mm). However, the other dilutions did not differ statistically from plants irrigated with supply water. This demonstrates that the salinity of the irrigation water negatively affects plant growth due to the specific effects of ions and the osmotic effect, which delay cell expansion and division, leading to negative consequences for photosynthetic rates and impairing the physiological and biochemical processes of plants.^36,37^ As a result, stem diameter is also reduced.

### 3.3 Biomass Accumulation: Leaf Dry Mass (LDM), Stem Dry Mass (SDM), Root Dry Mass (RDM), Total Dry Mass (TDM), and the Dickson Quality Index (DQI)

According to the analysis of variance (Table 4), a significant effect of the interaction between the factors oilfield produced water (OPW) × salicylic acid (SA) concentrations was observed on leaf dry mass (LDM), stem dry mass (SDM), total dry mass (TDM), and the Dickson Quality Index (DQI). The interaction between OPW × Barbados cherry genotypes (BCg) also affected LDM and SDM, and the interaction between SA × BCg affected LDM. Additionally, there were significant isolated effects of the factors OPW, SA, and BCg on root dry mass (RDM) of Barbados cherry plants, as well as an isolated effect of the factor BCg on the DQI.

**Table 4:** Summary of the Analysis of Variance for Leaf Dry Mass (LDM), Stem Dry Mass (SDM), Root Dry Mass (RDM), Total Dry Mass (TDM), and the Dickson Quality Index (DQI) of Barbados Cherry Genotypes under Irrigation with Oilfield Produced Water (OPW) Dilutions and Different Salicylic Acid (SA) Concentrations

	mean squares				
source of variation	LDM	SDM	RDM	TDM	DQI
oilfield produced water (OPW)	0.59^c^	0.19^a^	4.45^c^	9.78^c^	0.08^c^
salicylic acid concentrations (SA)	0.38^c^	0.09^a^	0.94^b^	2.81^c^	0.04^c^
linear regression	0.02^a^	0.25^a^	0.64^a^	2.13^a^	0.02^a^
quadratic regression	1.03^c^	0.001^a^	1.70^b^	5.54^c^	0.09^c^
Barbados cherry genotypes (BCg)	0.002^a^	2.79^c^	4.16^c^	0.18^a^	0.09^c^
interaction (OPW × SA)	0.25^c^	0.27^c^	0.58^a^	2.02^c^	0.02^b^
interaction (OPW × BCg)	0.18^b^	0.54^c^	0.15^a^	0.69^a^	0.01^a^
interaction (SA × BCg)	0.41^c^	0.03^a^	0.17^a^	0.92^a^	0.02^a^
interaction (OPW × SA × BCg)	0.12^a^	0.18^a^	0.75^a^	1.75^a^	0.03^a^
blocks	1.77^c^	3.16^c^	10.49^c^	39.66^c^	0.14^c^
source of variation	17.77	16.18	16.96	11.13	15.92

a Not significant.

b Significant at *p* ≤ 0.01.

c Significant at *p* ≤ 0.05; CV: coefficient of variation.

In the analysis of the interactive effect of oilfield produced water (OPW) dilutions and salicylic acid (SA) concentrations on leaf dry mass (LDM) (Figure 4A), a quadratic behavior was observed for the OPW-D3, OPW-D4, and OPW-D5 dilutions, with the highest values (1.69, 1.83, and 1.67 g) obtained at SA concentrations of 1.0, 1.2, and 1.2 mM, respectively. For the OPW-D1 dilution, there was a linear increasing response of 5.76% per unit increase in SA concentration, resulting in a 13.82% increase in LDM in plants receiving the highest SA dose compared to those that did not receive SA. This indicates that salicylic acid, at appropriate concentrations, acts on physiological processes such as photosynthesis, increasing biomass production,^38^ as well as mitigating saline stress,^39^ to which the plants were exposed in the OPW-D3, OPW-D4, and OPW-D5 treatments.

**Figure 4:**
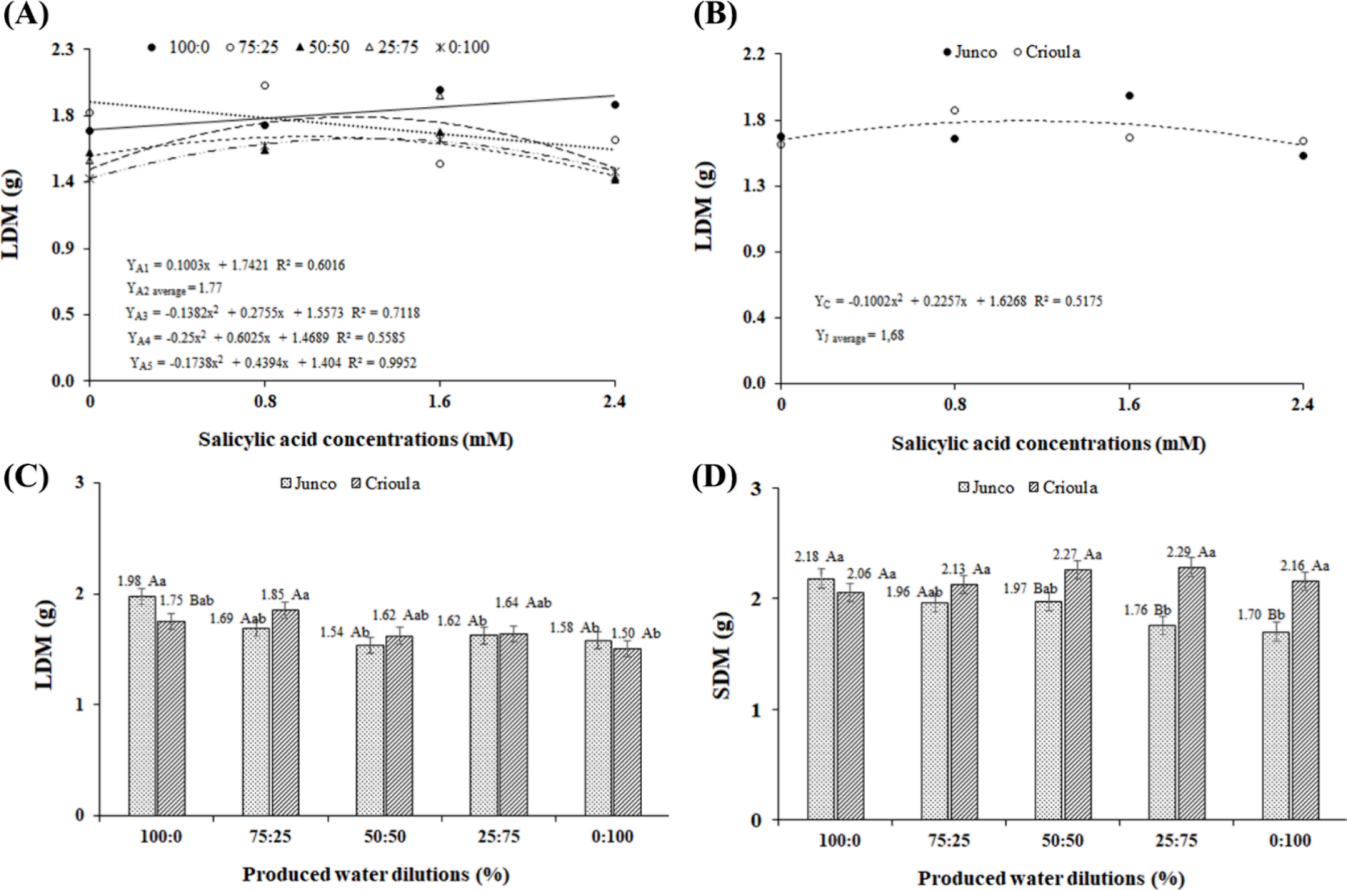
Leaf dry mass (LDM) as a function of the interaction between the factors oilfield produced water (OPW) and salicylic acid (SA) concentrations (A) and the interaction between SA and Barbados cherry genotypes (BCg) (B); and LDM (C) and stem dry mass (SDM) (D) as a function of the interaction between the factors OPW and BCg. Dilutions from left to right: D1 (100% supply water (SW)­100% SW), D2 (75% SW + 25% OPW), D3 (50% SW + 50% OPW), D4 (25% SW + 75% OPW), and D5 (100% OPW). Means with the same letters do not differ from each other according to Tukey’s test (*p* ≤ 0.01 for LDM and *p* ≤ 0.05 for SDM).

Through the breakdown of the interaction between the factors SA × BCg for the variable LDM, a significant effect was observed only for the *Crioula* genotype. According to the regression equation (Figure 4B), a quadratic behavior was observed, with the highest LDM value (1.75 g) in seedlings under an SA concentration of 1.1 mM. This is likely due to salicylic acid promoting the production of photosynthetic pigments and photoassimilates, thereby enhancing shoot growth^40^ and, consequently, increasing biomass.

The interaction between the factors OPW × BCg also affected LDM. According to the mean comparison test (Figure 4C), the *Junco* genotype under irrigation with OPW-D1 had the highest LDM value (1.98 g), although it did not differ statistically from OPW-D2 and was 20.2% higher than plants under OPW-D5. For the *Crioula* genotype, the highest LDM (1.85 g) was observed in plants under OPW-D2, which did not differ statistically from the other OPW dilution treatments, indicating greater tolerance of this genotype to saline stress in terms of LDM.

The interaction between OPW × BCg affected stem dry mass (SDM), promoting a significant difference between genotypes in the OPW-D3, OPW-D4, and OPW-D5 dilutions (Figure 4D). The *Crioula* genotype achieved the highest value (2.29 g) in seedlings under the OPW-D4 dilution, which did not differ statistically from the other OPW dilutions. The SDM of the *Junco* genotype reached the highest value (2.18 g) when irrigated with supply water (OPW-D1), but it did not differ statistically when subjected to the OPW-D2 and OPW-D3 dilutions. Based on the results, it is inferred that the *Crioula* genotype, as observed for LDM, showed greater tolerance when exposed to the salinity of the irrigation water, likely due to its genetic predisposition to be more resistant to salinity. Plant tolerance to salinity varies not only between species but also between genotypes of the same species and across developmental stages.^41,42^

The stem dry mass of the seedlings was also affected by the interaction between OPW × SA. In plants irrigated with OPW-D2 and OPW-D3, there was a linear decreasing effect of 8.44 and 4.96% per unit increase in SA concentration, respectively, resulting in reductions of 20.25 and 11.92% in plants subjected to the highest SA concentration compared to those that did not receive SA. In plants under OPW-D4, a quadratic behavior was observed according to the regression equation, with the highest value (2.18 g) obtained at an SA concentration of 1.3 mM (Figure 5A). This suggests that the decrease in SDM in plants under higher salinity levels is related to the osmotic effect caused by excess salts in the water, which alters ionic and osmotic homeostasis and reduces growth, resulting in lower biomass accumulation.^43^ However, it is observed that plants receiving an adequate SA concentration (1.3 mM) can increase SDM production even under irrigation with the OPW-D4 dilution (25% SW + 75% OPW), i.e., water with an electrical conductivity of 2.63 dS m^–1^, due to salicylic acid promoting plant metabolism and mitigating the effects of saline stress.^44^

**Figure 5:**
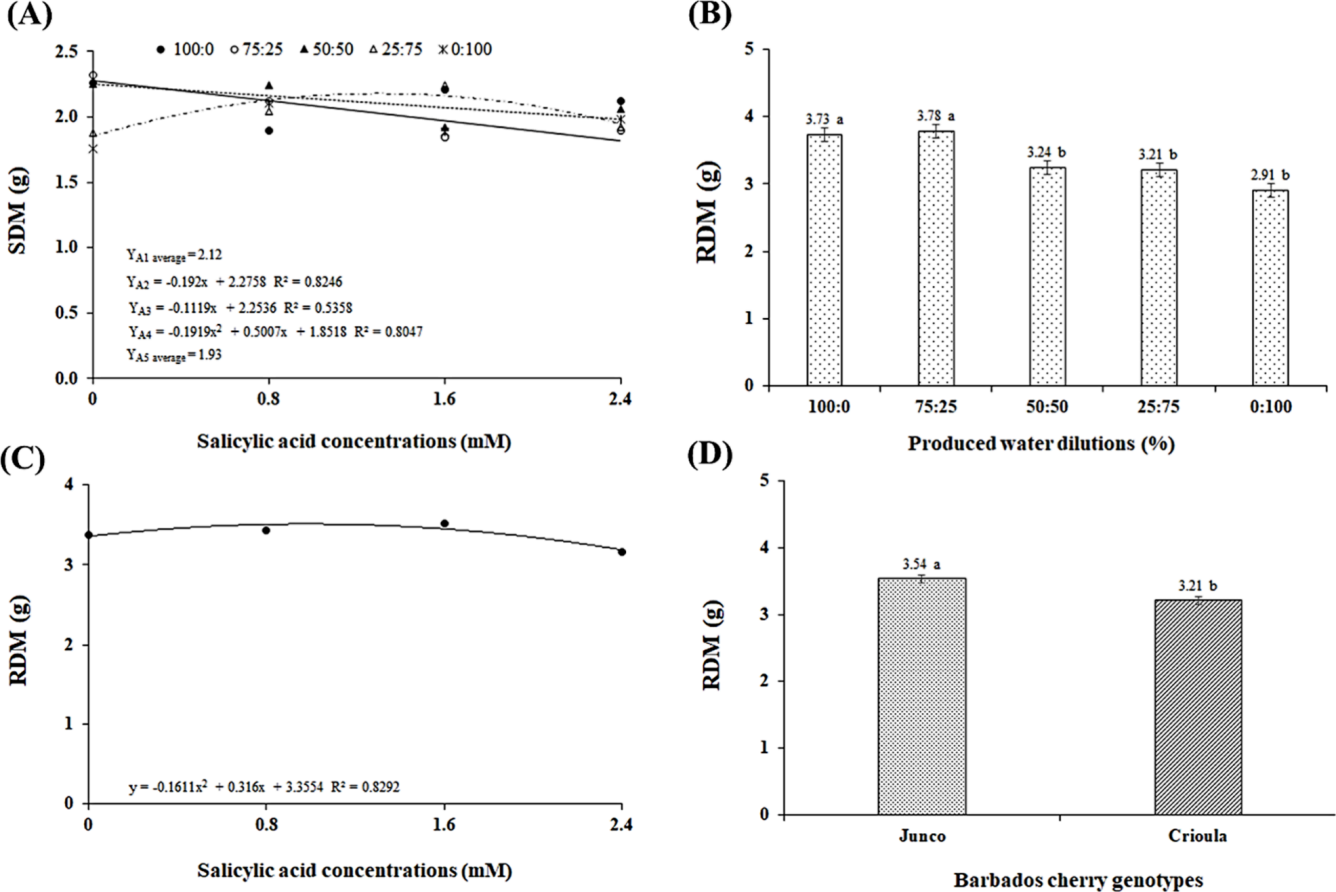
Stem dry mass (SDM) (A), total dry mass per plant (TDM) (B), Dickson Quality Index (DQI) (C), as a function of the interaction between the factors oilfield produced water (OPW) and salicylic acid (SA) concentrations; and DQI of the Barbados cherry genotypes (D). Dilutions from left to right: D1 (100% supply water (SW)), D2 (75% SW + 25% OPW), D3 (50% SW + 50% OPW), D4 (25% SW + 75% OPW), and D5 (100% OPW). Means with the same letters do not differ from each other according to Tukey’s test (*p* ≤ 0.05).

The root dry mass (RDM) of Barbados cherry was significantly affected by the OPW dilutions. According to the mean comparison test (Figure 5B), seedlings under the OPW-D2 dilution (75% SW + 25% OPW) had the highest RDM, although it did not differ statistically from OPW-D1 and was 23.01% higher than the RDM of seedlings under OPW-D5. This indicates that the root system was more sensitive to the effects of OPW salts, likely due to changes in the osmotic potential of the soil solution,^45^ causing the plant to expend more energy on water and nutrient uptake, thereby compromising growth.

The increasing concentrations of salicylic acid (SA) affected root dry mass (RDM). According to the regression equation (Figure 5C), a quadratic behavior was observed, with RDM increasing up to an SA concentration of 1.0 mM, which promoted the highest RDM (3.51 g). This is likely because salicylic acid, when applied at low concentrations, aids in the formation of adventitious roots and reduces the activity of the enzyme regulating indoleacetic acid homeostasis.^46^

The Barbados cherry genotypes differed statistically in terms of RDM. As illustrated in Figure 5D, the highest value (3.54 g) was observed in seedlings of the *Junco* genotype, representing a 9.32% superiority compared to the *Crioula* genotype, likely due to the genetic variability of the plant materials.

There was a significant response to the interaction between OPW × SA for total dry mass (TDM). According to the regression equations (Figure 6A), a quadratic response was observed for the OPW-D3, OPW-D4, and OPW-D5 dilutions, with the highest values of 7.75, 7.40, and 6.74 g, respectively, in seedlings under SA concentrations of 0.7, 1.4, and 1.1 mM. This suggests that the application of salicylic acid mitigated the effects of saline stress from oilfield produced water on Barbados cherry seedlings due to its role as a signaling molecule for biotic or abiotic stress^47^ and its ability to enhance CO_2_ assimilation rates and instantaneous water-use efficiency.^48^ For the OPW-D2 dilution, the behavior was linearly decreasing, with a reduction of 7.18% per unit increase in SA concentration, resulting in a 17.24% decrease in plants subjected to the highest SA concentration compared to those that did not receive SA. Thus, it is observed (Figure 6A) that higher SA concentrations, combined with increased salinity of the produced water, negatively affected TDM. This is likely attributed to the plant expending energy to osmotically adjust by accumulating sugars, organic acids, and ions in the vacuole, energy that could otherwise be used for biomass production.^49^

**Figure 6:**
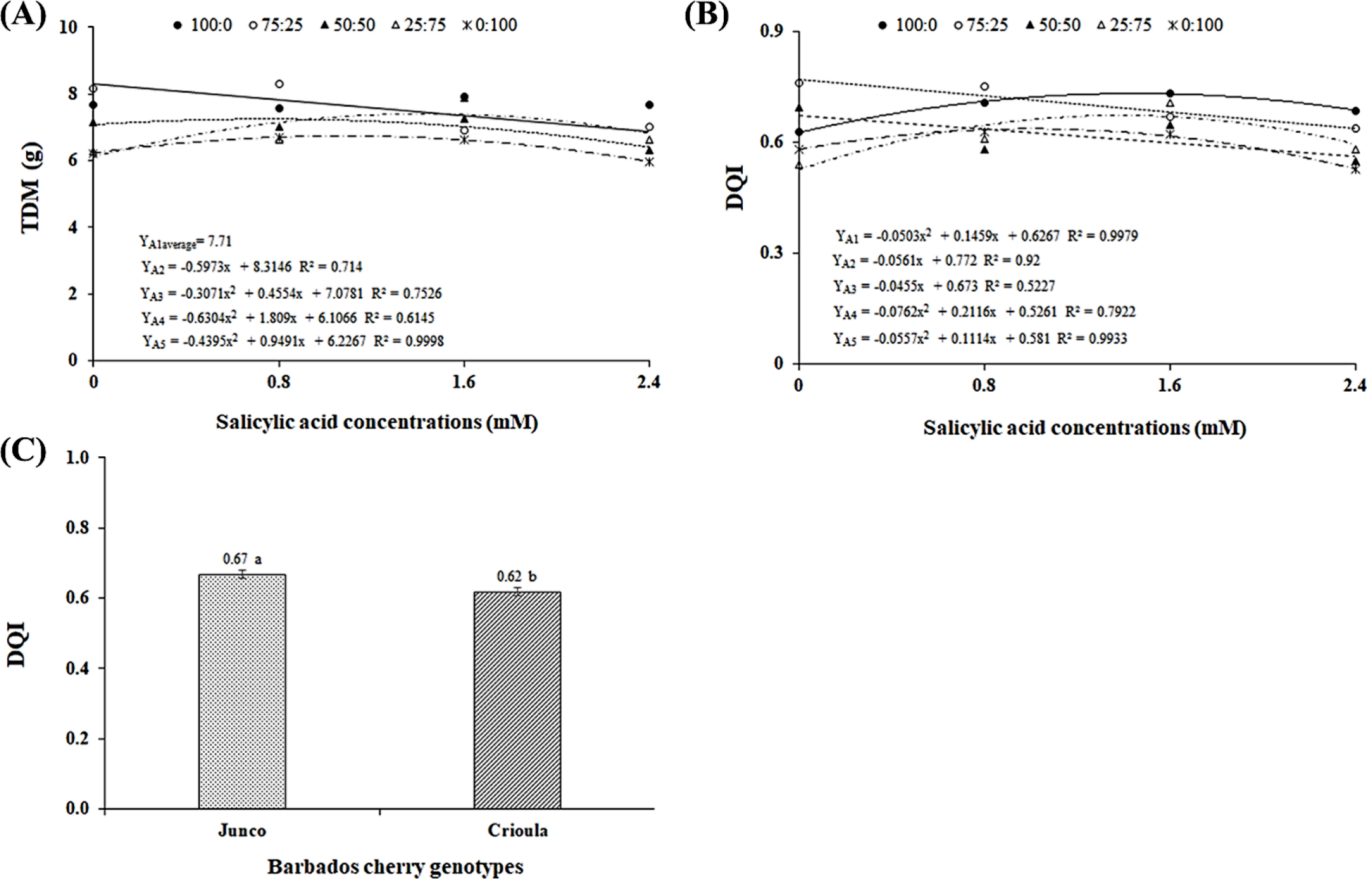
Root dry mass (RDM) as a function of oilfield produced water dilutions (A), salicylic acid concentrations (B), and different Barbados cherry genotypes (C). Dilutions from left to right: D1 (100% supply water (SW)), D2 (75% SW + 25% OPW), D3 (50% SW + 50% OPW), D4 (25% SW + 75% OPW), and D5 (100% OPW). Means with the same letters do not differ from each other according to Tukey’s test (*p* ≤ 0.05).

The Dickson Quality Index (DQI) was affected by the interaction between OPW × SA. According to the regression equations (Figure 6B), seedlings under the OPW-D2 and OPW-D3 dilutions showed a linear decreasing response of 7.27 and 6.76% per unit increase in SA concentration, respectively, resulting in reductions of 17.44 and 16.23% in plants under the highest SA concentration compared to those that did not receive SA. For seedlings under OPW-D1, OPW-D4, and OPW-D5, a quadratic behavior was observed, with the highest DQI values of 0.73, 0.67, and 0.64 obtained at SA concentrations of 1.5, 1.4, and 1.0 mM, respectively. This indicates, in yet another variable, that the application of SA at concentrations below 1.6 mM can mitigate the saline stress^50^ caused by OPW. However, at higher SA concentrations, an antagonistic effect on the plants may occur due to reduced translocation of SA to the aerial parts, potentially disrupting enzymatic activity and causing damage to the photosystem and growth.^51^

Furthermore, it is observed (Figure 6B) that Barbados cherry seedlings irrigated with OPW dilutions, with electrical conductivity ranging from 0.47 (OPW-D1) to 3.52 dS m^–1^ (OPW-D5), exhibited a DQI greater than 0.53. This indicates the tolerance of the plant materials to OPW salts and good seedling quality, making them suitable for transplanting to field conditions. According to Oliveira et al.,^52^ seedlings are classified as high quality when they exhibit a DQI greater than 0.2.

The Barbados cherry genotypes also differed significantly in terms of the Dickson Quality Index (Figure 6C). According to the mean comparison test, the DQI of the *Junco* genotype surpassed that of the *Crioula* genotype by 7.46%. This superiority suggests that seedlings of this genotype have a more balanced ratio between the shoot and root system, which is crucial for the successful establishment of plants after field transplantation.

The Dickson Quality Index (DQI) is a vital indicator of the quality and potential performance of fruit seedlings, including Barbados cherry. It integrates morphological and physiological parameters, such as total dry mass (TDM), root dry mass (RDM), and shoot-to-root ratios, to predict seedling vigor, survival, and growth after transplantation.^53,54^ In the context of this study, the DQI was essential for evaluating the quality of Barbados cherry seedlings irrigated with oilfield produced water (OPW) and treated with salicylic acid (SA), highlighting the superiority of the *Junco* genotype, which exhibited a DQI 7.46% higher than that of the *Crioula* genotype.

The importance of the DQI lies in its predictive capacity and correlation with essential growth metrics. Seedlings with higher DQI values, such as those observed in this study (DQI > 0.60), are associated with better survival and growth rates under field conditions, especially in adverse environments such as semiarid regions.^53,55^ This is particularly relevant for Barbados cherry cultivation, where adaptation to abiotic stresses, such as salinity and water deficit, is critical for the successful establishment of plants after transplantation.

The selection of seedlings with high DQI, such as those of the *Junco* genotype, allows for the preselection of plant materials with superior growth characteristics and greater resistance to environmental stresses.^53,54^ This strategy is especially important in agricultural systems that use lower-quality water, such as OPW, as it ensures that seedlings are more likely to adapt quickly to field conditions, reducing post-transplantation losses and increasing the efficiency of water and nutrient use.

This adaptability not only reduces dependence on conventional water resources but also contributes to the reduction of improper OPW disposal, promoting more sustainable agricultural practices aligned with the United Nations Sustainable Development Goals (SDGs), particularly SDG 2 (Zero Hunger and Sustainable Agriculture) and SDG 6 (Clean Water and Sanitation).

## 4 Conclusions

There are provisions for the use of water produced oilfield for the production of Barbados cherry seedlings in the semiarid region of Brazil with the application of hydrogen peroxide as an attenuator, contributing to environmental preservation and sustainability.

The average salicylic acid concentration of 1.3 mM demonstrated efficacy in mitigating the effects of saline stress from oilfield produced water (OPW) up to 2.63 dS m^–1^, promoting biomass production and the quality of Barbados cherry seedlings. Irrigation with the OPW-D3 dilution (50% supply water +50% OPW) proved favorable for the morphophysiology of seedlings of the *Junco* and *Crioula* genotypes, standing out as a viable strategy for the sustainable use of OPW in agriculture. The *Junco* genotype exhibited the best seedling quality, while the *Crioula* genotype showed greater tolerance to saline stress, highlighting the importance of selecting adapted genotypes for cultivation under adverse conditions.

In addition to agronomic benefits, this study contributes to the discussion on the reuse of oil industry waste, reducing improper OPW disposal and promoting water conservation in semiarid regions. Salicylic acid could be applied via nebulization since the amount of SA applied is very small, making product loss due to drift negligible. However, future evaluation of the large-scale use of OPW for irrigation should consider potential effects on soil and groundwater salinity, as well as risks to human health and the ecosystem.

Long-term studies are needed to assess the cumulative impacts of OPW on soil quality, microbiology, and food safety. The combination of OPW with SA may represent a promising solution for agriculture in water-scarce regions, provided that appropriate monitoring and management practices are adopted. Furthermore, studies evaluating the safety impacts of OPW on food crops are also scarce, particularly regarding the likely presence of countervailing factors on human and animal health.

This research reinforces the importance of interdisciplinary approaches to address environmental challenges, integrating knowledge from irrigation, ecotoxicology, and plant physiology. Future studies should explore the application of these strategies in other crops and ecosystems, as well as investigate the biochemical and molecular mechanisms involved in SA-mediated mitigation of saline stress. In this way, the study not only advances scientific understanding of the topic but also provides practical insights for the sustainable management of water resources and the promotion of food security in a context of climate change and anthropogenic pressures.

## Data Availability

All data generated or analyzed during this study are included in this published article.

## Abbreviations

SDS: United Nations Sustainable Development Goals
SDG 2: Zero Hunger and Sustainable Agriculture
OPW: oilfield produced water
SA: salicylic acid
JA: jasmonic acid
ET: ethylene
ABA: abscisic acid
SW: supply water
EC: electrical conductivity
T: cation exchange capacity
V: base saturation
m: aluminum saturation
DAT: days after transplanting
CP: commercial product
w/v: weight/volume
NL: number of leaves
PH: plant height
SD: stem diameter
IRGA: infrared carbon dioxide analyzer
Ci: intercellular CO2 concentration
*gs*: stomatal conductance
*A*: CO_2_ assimilation rate
LDM: leave dry mass
SDM: stem dry mass
RDM: root dry mass
TDM: total dry mass
DQI: Dickson Quality Index
ANOVA: analysis of variance
GA: genotypes
CV: coefficient of variation
